# Cost-Effectiveness of Digital Mental Health Versus Usual Care During Humanitarian Crises in Lebanon: Pragmatic Randomized Trial

**DOI:** 10.2196/55544

**Published:** 2024-05-29

**Authors:** Racha Abi Hana, Jinane Abi Ramia, Sebastian Burchert, Kenneth Carswell, Pim Cuijpers, Eva Heim, Christine Knaevelsrud, Philip Noun, Marit Sijbrandij, Mark van Ommeren, Edith van’t Hof, Ben Wijnen, Edwina Zoghbi, Rabih El Chammay, Filip Smit

**Affiliations:** 1 Clinical, Neuro- and Developmental Psychology Department Vrije Universiteit Amsterdam Amsterdam Netherlands; 2 National Mental Health Programme Ministry of Public Health Beirut Lebanon; 3 Division of Clinical Psychological Intervention Department of Education and Psychology Freie Universität Berlin Berlin Germany; 4 Department of Mental Health and Substance Use World Health Organization Geneva Switzerland; 5 International Institute for Psychotherapy Babeş-Bolyai University Cluj-Napoca Romania; 6 Institute of Psychology University of Lausanne Lausanne Switzerland; 7 Department of Psychology University of Zurich Zurich Switzerland; 8 Centre of Economic Evaluation and Machine Learning Trimbos Institute (Netherlands Institute of Mental Health and Addiction) Utrecht Netherlands; 9 Country Office for Lebanon World Health Organization Beirut Lebanon; 10 Faculty of Medicine Psychiatry Department Saint Joseph University Beirut Lebanon; 11 Department of Epidemiology and Biostatistics Amsterdam University Medical Centers Amsterdam Netherlands; 12 Department of Mental Health and Prevention Trimbos Institute (Netherlands Institute of Mental Health and Addiction) Utrecht Netherlands

**Keywords:** depression, internet-based intervention, economic evaluation, Lebanese, Syrian, digital mental health, digital health, mental health, usual care, Lebanon, anxiety, stress-related disorders, treatment, symptoms, large randomized controlled trial, effectiveness

## Abstract

**Background:**

There is evidence from meta-analyses and systematic reviews that digital mental health interventions for depression, anxiety, and stress-related disorders tend to be cost-effective. However, no such evidence exists for guided digital mental health care in low and middle-income countries (LMICs) facing humanitarian crises, where the needs are highest. Step-by-Step (SbS), a digital mental health intervention for depression, anxiety, and stress-related disorders, proved to be effective for Lebanese citizens and war-affected Syrians residing in Lebanon. Assessing the cost-effectiveness of SbS is crucial because Lebanon’s overstretched health care system must prioritize cost-effective treatment options in the face of continuing humanitarian and economic crises.

**Objective:**

This study aims to assess the cost-effectiveness of SbS in a randomized comparison with enhanced usual care (EUC).

**Methods:**

The cost-effectiveness analysis was conducted alongside a pragmatic randomized controlled trial in 2 parallel groups comparing SbS (n=614) with EUC (n=635). The primary outcome was cost (in US $ for the reference year 2019) per treatment response of depressive symptoms, defined as >50% reduction of depressive symptoms measured using the Patient Health Questionnaire (PHQ). The secondary outcome was cost per remission of depressive symptoms, defined as a PHQ score <5 at last follow-up (5 months post baseline). The evaluation was conducted first from the health care perspective then from the societal perspective.

**Results:**

Taking the health care perspective, SbS had an 80% probability to be regarded as cost-effective compared with EUC when there is a willingness to pay US $220 per additional treatment response or US $840 per additional remission. Taking the wider societal perspective, SbS had a >75% probability to be cost-saving while gaining response or remission.

**Conclusions:**

To our knowledge, this study is the first cost-effectiveness analysis based on a large randomized controlled trial (n=1249) of a guided digital mental health intervention in an LMIC. From the principal findings, 2 implications flowed, from the (1) health care perspective and (2) wider societal perspective. First, our findings suggest that SbS is associated with greater health benefits, albeit for higher costs than EUC. It is up to decision makers in health care to decide if they find the balance between additional health gains and additional health care costs acceptable. Second, as seen from the wider societal perspective, there is a substantial likelihood that SbS is not costing more than EUC but is associated with cost-savings as SBS participants become more productive, thus offsetting their health care costs. This finding may suggest to policy makers that it is in the interest of both population health and the wider Lebanese economy to implement SbS on a wide scale. In brief, SbS may offer a scalable, potentially cost-saving response to humanitarian emergencies in an LMIC.

**Trial Registration:**

ClinicalTrials.gov NCT03720769; https://clinicaltrials.gov/ct2/show/NCT03720769

**International Registered Report Identifier (IRRID):**

RR2-10.2196/21585

## Introduction

In the last decade, Lebanon registered an influx of around 1 million Syrian displaced people [1], in addition to approximately 0.5 million unregistered refugees. They are at considerable risk of developing mental disorders such as depression, anxiety, and posttraumatic stress disorder and often in need of mental health services [2]. Meanwhile, Lebanon’s own population suffered from a series of overlapping humanitarian and economic crises (ongoing political turmoil, hyperinflation, the COVID-19 pandemic, the Beirut port blast), which undermined population mental health [3] and exacerbated the pressure on Lebanon’s already overstretched health care system. As a result of these emergencies, Lebanese health care workers have been put under immense pressure, and many sought to leave the country to work overseas [4].

Considering the crises-related needs, transformations were required in line with Lebanon’s national mental health strategy, in which one of its objectives was to scale-up digital self-help programs for priority conditions such as depression, anxiety, and posttraumatic stress in a cost-effective way [5]. To this end, the World Health Organization (WHO) helped to develop, test, and implement a guided e-mental health intervention for depression among Lebanese citizens, displaced Syrians, and other people residing in Lebanon [6-10]. The intervention is called Step-by-Step (SbS) and can be downloaded on digital devices. SbS consists of 5 sessions based on evidence-based psychological treatments, primarily behavioral activation. It is delivered with the aid of nonspecialist helpers trained and supervised to provide participants with guidance via messaging or by phone [6].

Elsewhere, we demonstrated that the SbS intervention is effective in reducing depression, anxiety, and posttraumatic stress and in improving personal and social functioning and subjective well-being in Lebanese citizens, Syrians, and other populations residing in Lebanon [11,12]. The objective of this paper was to report on the cost-effectiveness of offering the effective SbS intervention in Lebanon. This study is among the first to evaluate the cost-effectiveness of scaling up a response to crises using a digital intervention in low- and middle-income countries.

## Methods

### Study Design

The study was designed as a cost-effectiveness analysis alongside a pragmatic randomized controlled trial with 2 parallel groups comparing SbS with enhanced usual care (EUC) with assessments at baseline (t0); 8 weeks post baseline (t1), which was after completion of the intervention; and 20 weeks post baseline (t2), hence 3 months after conclusion of the intervention. A study protocol [10], pilot study [8], and feasibility trial [9] have been published, and effectiveness studies showed positive clinical effects on depression, disability, and symptoms of anxiety and posttraumatic stress [11,12].

### Ethics Approval

The WHO (ERC.0002797) and Saint Joseph’s University in Beirut (CEHDF862) Ethical Review Committees provided medical ethics approval.

### Participants

Participants were Lebanese citizens, displaced Syrians in Lebanon, and other people residing in Lebanon. Participants were recruited via social media, online advertisements, and outreach activities. Participants could download the SbS app for iOS and Android or visit its web version, which provided information about the intervention and the study and included a screener for eligibility. To be included in the study, participants had to (1) be aged 18 years or older, (2) reside in Lebanon, (3) be able to speak and understand Arabic or English, (4) have access to a device connected to the internet, (5) score higher than 10 on the Patient Health Questionnaire (PHQ-9) [13] for depressive symptom severity, and (6) score higher than 16 on the World Health Organization Disability Assessment Scale Schedule-12 (WHODAS) for impaired functioning [14]. The exclusion criterion was imminent risk of suicide, in which case the participant received psychoeducation and was directed to the national suicide prevention lifeline. Consenting participants who fulfilled the inclusion criteria were invited to complete the baseline questionnaire. As an incentive, those who completed the questionnaires received a US $20 phone credit.

### Randomization and Masking

Eligible and consenting participants were randomized to either the SbS intervention or EUC using automated permuted block randomization with a 1:1 allocation ratio within blocks of random length between 2 and 8. Randomization was stratified for nationality: Syrians in Lebanon versus Lebanese citizens and other people residing in Lebanon. The randomization algorithm was built into the SbS app and was not accessible by the research team.

### Interventions

SbS was a guided digital health intervention to alleviate symptoms of depression, anxiety, and posttraumatic stress [6]. The intervention was based on evidence-based therapeutic techniques. The main therapeutic technique was behavioral activation with additional psychoeducation, stress management, positive self-talk, gratitude practice, reinforcing social support, and relapse prevention [6]. Weekly support to users was provided by phone or messaging by trained nonspecialists called “e-helpers” [9]. The e-helpers were trained over 5 days and continued to receive weekly group supervision by a clinical supervisor as well as individual supervision when needed [11,12]. e-Helpers could only begin their job after passing a competency examination subsequent to the completion of their training. Using a treatment fidelity checklist [11], fidelity checks found minor deviations from the treatment plan, such as when e-helpers skipped practice activities or did not go through the story with users fully [11].

EUC consisted of a psychoeducational message on the SbS app (similar to the first SbS session) and a list of primary health care centers available in different areas in Lebanon. Staff in these health centers were trained in screening, detecting, and managing mental health conditions [15].

### Outcome Measures

The central outcome was the PHQ-9 [13]. The PHQ-9 is a 9-item instrument measuring severity of depression, with a cutoff score >10 indicating moderate to severe depression, which has also been validated in Lebanon [16]. For clinical and economic interpretation, the PHQ-9 was converted to treatment response and remission. Response was defined as an improvement of at least 50% between t0 and t2 in depressive symptom severity as measured by the PHQ-9. Remission was defined as a participant’s PHQ-9 score below 5 at t2 [17].

### Resource Use and Costs

#### Questionnaires

Costs stemming from health care uptake and productivity losses were collected using a Lebanese Resource Use questionnaire that was based on both the Trimbos and iMTA (Institute of Medical Technology Assessment) Cost questionnaire for Psychiatric illness (TiC-P) [18], which has good reliability and validity [19], and the cross culturally validated Client Service Receipt Inventory (CSRI) [20] adapted and piloted for use in Lebanon [9]. The Lebanese Resource Use questionnaire was programmed into the SbS app as a self-report questionnaire.

All costs are expressed in US $ for the year 2019 when the study was carried out and when, according to the World Bank, the average exchange rate for US $1 was LBP 1507.50.

#### Cost of SbS

In the year 2019, the personnel cost of offering SbS amounted to US $59,520 (consisting of the gross annual salaries of a 0.2 full-time equivalent [FTE] clinical supervisor, 1 FTE coordinator, 1 FTE senior e-helper, and 2 FTE e-helper). The annual nonpersonnel cost of offering SbS was US $62,800 (US $36,000 for hosting, maintaining, and periodically upgrading the digital intervention; US $14,800 for renting the office, equipment, and overhead; and US $2000 for advertising). The total personnel and nonpersonnel costs of operating SbS was therefore US $59,520 + $62,800 = $122,320. SbS can serve 4700 users in a year. Therefore, the per-user costs of SbS was US $122,320/4700 = US $26.

#### Cost of EUC

People randomized to EUC received an online psychoeducational message derived from the first session of the SbS intervention. They also received a list of primary health care facilities with nonspecialized staff trained in the Mental Health Gap Action Programme [15]. The per-user cost of this online message was next to nothing (US $0.01) and was ignored in the subsequent analyses.

#### Cost of Health Care Utilization and Productivity Costs

Table 1 reports the cost prices per unit health care, such as a visit to a general practitioner, session with a psychologist, or day in a mental ward.

Costs can be regarded from the health care perspective and from the societal perspective. Taking the health care perspective, only the direct medical costs were considered, stemming from the contacts of participants with health services. Costs were evaluated first from the health care perspective and second from the broader societal perspective, thus adding the costs stemming from productivity losses to health care costs. Productivity losses occur when a person stays absent from work (absenteeism) as well as when a person does not feel well, tries to work anyway but is less productive (presenteeism), resulting in work cutback.

**Table 1:** Unit cost prices in US $ (2019 price level).

Item		Unit	Unit cost price (US $), mean	Range	Source
**Intervention**					
	Step by Step	Usage	26.00	—^a^	Estimate 1^b^
	EUC^c^ psychoeducation	Online message	0.01	—	Estimate 1
**Primary care**					
	General practitioner (GP)	Contact	23.66	7.32^d^-40.00^e^	GPs
	Nurse	Contact	1.83	—	Ministry PH^f^
	Social worker	Contact	2.59	—	Ministry PH^f^
**Outpatient care**					
	Psychiatrist	Consult	56.06	24.62^d^-87.50^e^	Ministry PH^f^
	Neurologist	Consult	37.33	9.66^d^-65.00^e^	Ministry PH^f^
	Psychologist	Session	25.37	13.23^d^-37.50^e^	Ministry PH^f^
**Inpatient care**					
	Psychiatric ward	Day	325.00	150.00^d^-500.00^e^	Hospital
	Mental hospital	Day	150.00	150.00^e^	Hospital
**Emergency**					
	Ambulance trip	Transport	99.00	—	Red Cross
	Emergency room	Visit	48.75	25.00^d^-72.50^e^	Hospital
**Medication**					
	Antidepressants	DDD^g^	0.63	0.41-0.90	Estimate 2^h^
	Anxiolytics	DDD	0.36	0.23-0.55	Estimate 3^i^
	Hypnotics	DDD	0.39	0.23-0.55	Estimate 4^j^
**Productivity**					
	Paid work	Workday	32.09	16.66^k^-47.51^l^	Estimate 5^m^
	Unpaid work	Workday	7.82	—	Estimate 6^n^

^a^Not applicable.

^b^See the “Resource Use and Costs” section.

^c^EUC: enhanced usual care.

^d^Public.

^e^Private.

^f^Tariffs provided by the Ministry of Public Health (PH).

^g^DDD: daily defined dose.

^h^Average cost price per DDD of frequently prescribed antidepressants (escitalopram, sertraline, amitriptyline, clomipramine, venlafaxine).

^i^Average cost price per DDD of frequently prescribed anxiolytics (bromazepam, alprazolam, lorazepam, hydroxyzine, alprazolam).

^j^Average cost price per daily defined dose of hypnotics (zolpidem and melatonin).

^k^Syrian.

^l^Lebanese.

^m^Based on the Labour Market Assessment in Beirut and Mount Lebanon by the Agency for Technical Cooperation and Development [21] and indexed for the year 2019.

^n^Opportunity costs valued as the average per diem salary of domestic help in Lebanon in 2019.

### Base Case Analysis

The health economic evaluation was conducted in agreement with the Consolidated Health Economic Evaluation Reporting Standards (CHEERS) 2022 guideline for trial-based economic evaluations [22]. The costs are reported in US $ for the reference year 2019. The study’s time horizon was 20 weeks (ie, 5 months).

With a sample size of 1136, the study would be powered to detect a standardized mean difference of medium size (*d*≥0.50) between the conditions, as statistically significant at α≤.05 (2-tailed) and a power of (1 – β)≥0.90 while accounting for an expected dropout rate of 70%, which is typical for self-help interventions [23]. The study was powered to evaluate a clinical depression–related outcome but not for a health economic evaluation for which costs are typically associated with large standard errors. In other words, in this study, we could statistically test effect differences but not cost differences. Instead, health-economic inferences were not based on statistical hypothesis testing but on probabilistic medical decision-making techniques.

At each assessment (t0, t1, and t2), cost data were collected retrospectively from the last 4 weeks. Cumulative costs over the full trial duration of 20 weeks were computed using linear interpolation among the t0, t1, and t2 assessments. Incremental costs and incremental effects were computed as the difference of the cumulative costs and effects between the conditions. The incremental cost-effectiveness ratio (ICER) was computed as the cost difference over the effect difference: ICER = (C_1_-C_0_)/(E_1_-E_0_), where C and E are costs and effects and the subscripts 1 and 0 refer to the SbS and EUC conditions, respectively. The ICER is interpreted as the additional costs for gaining a treatment response and remission.

For intention-to-treat analysis, missing PHQ-9 scores were imputed using regression imputation. The regression imputation model included 2 types of predictors: predictors of outcome (societal costs, PHQ-9, gender, age, education, and WHO-5 Wellbeing as measured at t0) and predictors of missingness (randomization status, partner status, employment status, and WHODAS-12 at t0). The first set of predictors was included for predictive accuracy of the outcome, and the second type was included to better satisfy the missing-at-random assumption [24]. After imputation, the PHQ-9 score was converted into treatment response and remission.

**Figure 1:**
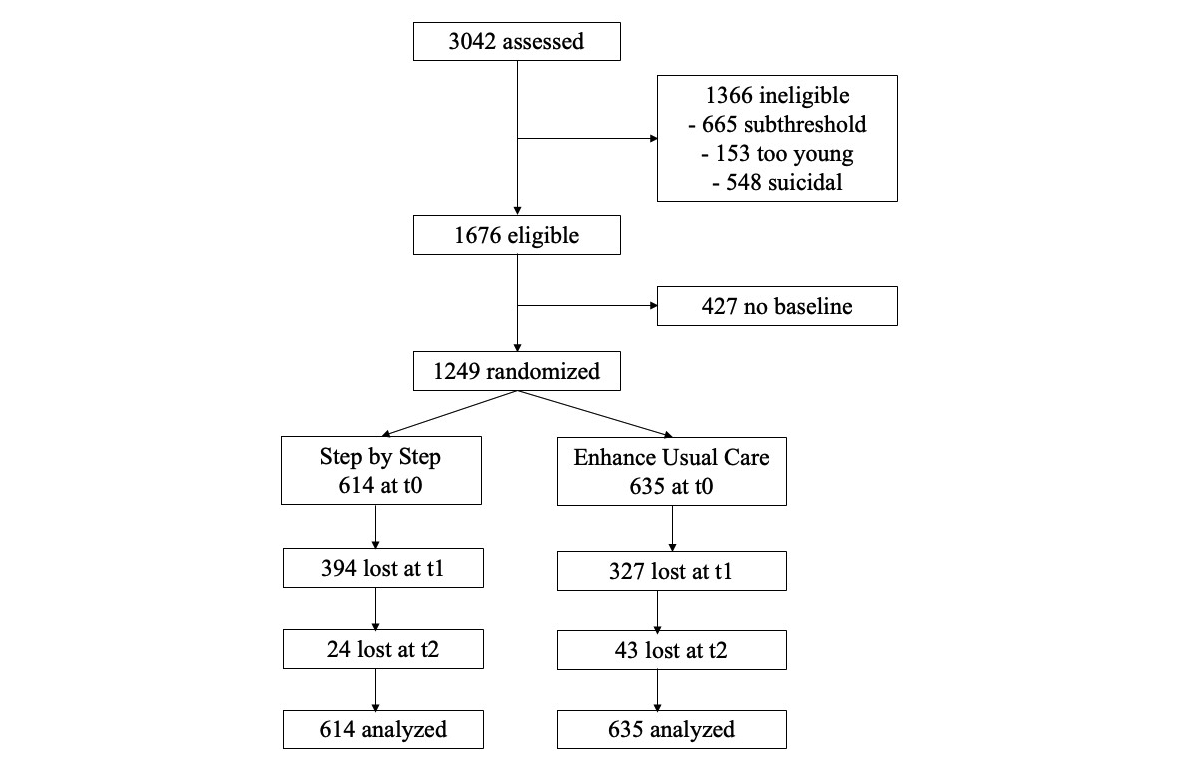
CONSORT (Consolidated Standards of Reporting Trials) flowchart. t0: baseline; t1: 8 weeks post baseline, which was after completion of the intervention; t2: 20 weeks post baseline.

To simultaneously evaluate both incremental costs and effects, seemingly unrelated regression equations (SURE) models were used. In the SURE model, baseline costs were included to adjust for a baseline imbalance because EUC had higher baseline costs than SbS. Since cost data were nonnormally distributed, nonparametric bootstraps (2500 times) were used for the SURE models. The bootstrapped SURE models helped to create 2 figures: (1) the ICER plane on which the simulated ICERs are plotted and (2) the acceptability curve. We return to these figures and their interpretation in the Results section.

### Sensitivity Analysis

The base case analysis had treatment response and depressive remission as outcomes. It was first conducted from the health care perspective, then it was repeated from the societal perspective. Missing observations due to dropout were imputed using regression imputation. In a preplanned sensitivity analysis, the base case analysis was repeated using multiple imputation with chained equations (MICE) using predictive mean matching to impute missing observations [25]. This was done to see how robust the results were under varying imputation strategies. All analyses were carried out in Stata 17.0 [26].

## Results

### Participants

Recruitment of the participants started December 9, 2019, and ended on July 9, 2020. In this 7-month period, 3042 persons were assessed for eligibility, 1676 met the inclusion criteria, and 1249 were randomized: 614 to SbS and 635 to EUC. Figure 1 shows the flow of participants through the trial.

At t1, dropout was 64.2% (394/614) in the SbS group and 51.5% (327/635) in the EUC group. At t2, these rates increased by an additional 24 and 43 participants, respectively, such that total dropout became 68.1% (418/614) in the SbS group and 58.3% (370/635) in the EUC group. Total dropout in the whole sample was 788/1249, or 63.1%. As indicated, a 70% dropout has to be expected in digital self-help interventions [23].

The demographic, clinical, and economic characteristics of the participants are listed in Table 2. It appears that randomization led to an even distribution of these variables over the conditions; however, societal costs appeared somewhat higher in the EUC group than the SbS group (Table 2).

**Table 2:** Characteristics of the sample at baseline (N=1249).

Characteristic			SbS^a^ (n=614)		EUC^b^ (n=635)		Total (N=1249)
Age (years), mean			29.0		29.1		29.1
**Gender, n (%)**							
	Female	415 (67.6)		393 (61.9)		808 (64.7)	
	Male	199 (32.4)		242 (38.1)		441 (35.3)	
**Marital status, n (%)**							
	Never married	275 (44.8)		289 (45.6)		564 (45.2)	
	Married	285 (46.4)		300 (47.2)		585 (46.8)	
	Other	54 (8.8)		46 (7.2)		100 (8)	
**Nationality, n (%)**							
	Lebanese	307 (50)		315 (49.6)		622 (49.8)	
	Syrian	275 (44.8)		283 (44.6)		558 (44.7)	
	Other	32 (5.2)		37 (5.8)		69 (5.5)	
**Education, n (%)**							
	Primary	138 (22.5)		128 (20.1)		266 (21.3)	
	Secondary	136 (22.2)		154 (24.3)		290 (23.2)	
	Vocational	238 (38.8)		261 (41.1)		499 (40)	
	Academic	102 (16.6)		92 (14.5)		194 (15.5)	
**Employment status, n (%)**							
	Employed	143 (23.3)		169 (26.6)		312 (25)	
	Homemaker	97 (15.8)		98 (15.4)		195 (15.6)	
	Student	106 (17.3)		107 (16.9)		213 (17.1)	
	Retired	1 (0.2)		4 (0.6)		5 (0.4)	
	Unemployed	267 (43.5)		257 (40.5)		524 (42)	
**Clinical characteristics, mean**							
	PHQ^c^ depression	16.4		16.4		16.4	
	WHODAS^d^ disability	33.0		33.1		33.0	
**Costs (last 4 weeks; US $)**							
	Health care costs	42		45		44	
	Societal costs	107		124		115	

^a^SbS: Step-by-Step.

^b^EUC: enhanced usual care.

^c^PHQ: Patient Health Questionnaire.

^d^WHODAS: World Health Organization Disability Assessment Scale.

### Cumulative Costs

Table 3 describes the costs in the last 4 weeks at the t0, t1, and t2 assessments and how these costs accumulated over the full 20-week period. Missing cost data at t1 and t2 due to dropouts were imputed; the per-user cost of SbS was not included.

Table 3 shows that the mean health care costs fluctuated per participant slightly over time. The cumulative health care costs were virtually the same in both groups (US $241 [SbS] vs US $243 [EUC]). However, the cumulative productivity costs ended up being lower with SbS (US $151) than with EUC (US $202), indicating that SbS helped to reduce productivity losses. This is also mirrored in the mean societal costs that were US $443 with EUC and US $393 with SbS. A detailed breakdown of health care and productivity costs is provided in Table S1 in Multimedia Appendix 1.

**Table 3:** Per-participant costs in US $ over time by condition, not including US $26 Step-by-Step (SbS) costs (N=1249).

Costs by group			t0^a^ (US $)		t1^b^ (US $)		t2^c^ (US $)		Cum (t0-t2) cost (US $; 95% CI)
**Health care**									
	EUC^d^	45		48		51		241 (203-279)	
	SbS	42		55		43		243 (209-276)	
**Productivity**									
	EUC	79		17		55		202 (176-229)	
	SbS	65		15		32		151 (129-172)	
**Societal**									
	EUC	124		65		105		443 (396-490)	
	SbS	107		70		75		393 (354-433)	

^a^Baseline.

^b^8 weeks post baseline, which was after completion of the intervention.

^c^20 weeks post baseline.

^d^EUC: enhanced usual care.

### Base Case Analysis

#### Incremental Costs

Taking the health care perspective, including the SbS costs of US $26 per recipient, the bootstrapped incremental health care costs averaged US $28 (95% CI –$22 to $78), suggesting that SbS costs somewhat more than EUC, but this was not statistically significant (bootstrap SE=25.44, *z*=1.10; *P*=.27). For context, US $28 would buy a single session with a psychologist in Lebanon in 2019.

Taking the societal perspective, the incremental costs averaged US –$24 (95% CI –$85 to $37), suggesting a small cost reduction favoring SbS over EUC, which was not statistically significant (bootstrap SE=31.15, *z*=–0.76; *P*=.45).

It is worth noting that there is a discrepancy between the incremental cost as seen from the health care perspective (US $28) and the incremental costs as seen from the societal perspective (US –$24). The cost reduction that comes into view when taking the societal perspective must be related to the greater productivity (less absenteeism and less presenteeism) among the recipients of SbS.

#### Incremental Effects

The incremental response rate was 0.23 (95% CI 0.18 to 0.27) favoring SbS in a statistically significant way (bootstrap SE=0.022, *z*=10.24; *P*<.001).

The incremental remission rate was 0.06 (95% CI 0.031 to 0.085) favoring SbS over EUC and was statistically significant (bootstrap SE=0.014; *z*=4.20; *P*<.001).

#### ICER Response: Health Care Perspective

**Figure 2:**
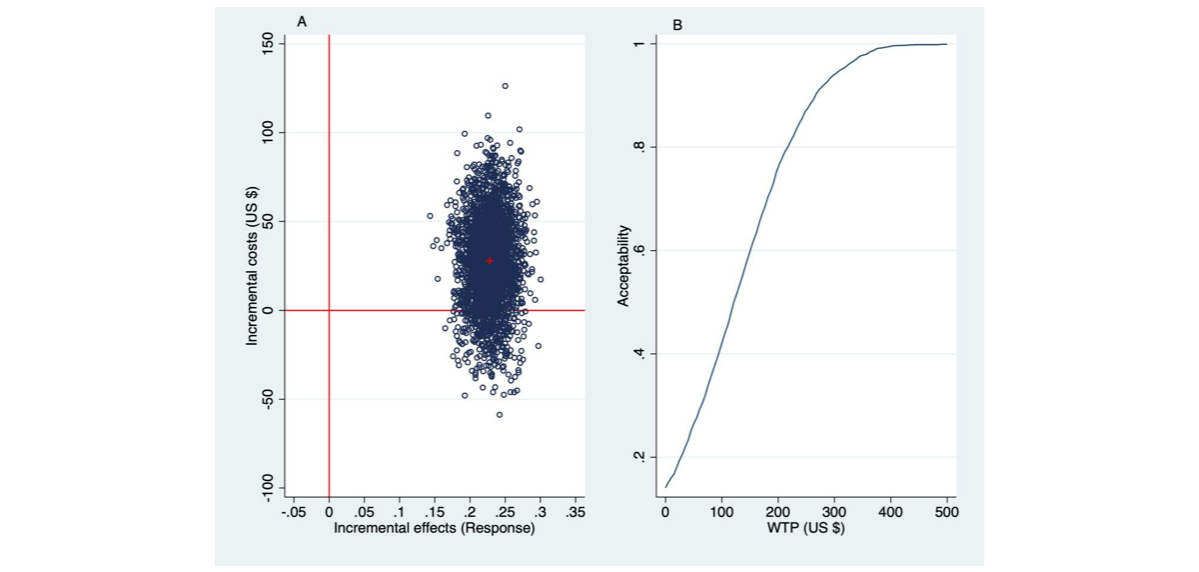
Health care perspective: incremental costs per additional Patient Health Questionnaire (9-item version) responder shown in a (A) cost-effectiveness plane and (B) acceptability curve (2500 bootstraps). WTP: willingness to pay.

Dividing the incremental health care costs of US $28 by the incremental response rate of 0.23 gives an ICER of US $28/0.23=$122. Thus, SbS costs US $122 more than EUC per treatment responder. It should be noted that the ICER of US $122 is an average and is surrounded by uncertainty, as depicted in Figure 2.

Figure 2A shows the uncertainty around the mean ICER as a scatter of simulated ICERs over the ICER plane. The ICER plane is divided into 4 quadrants, denoted North East (NE), North West (NW), South West (SW), and South East (SE). Of the bootstrapped ICERs, 86% appear in the NE quadrant of the ICER plane, indicating an 86% probability that better effects are obtained by SbS albeit for higher costs than EUC. The remainder of the simulated ICERs appear in the SE quadrant, indicating that health gains are achieved by SbS while cost reductions occurred compared with EUC. Thus, SbS generates a greater health gain albeit for more costs than EUC.

This begs the question about how much one is willing to pay to gain an additional treatment responder. The acceptability curve (Figure 2B) is now key to decision-making. The acceptability curve plots the probability that the intervention is deemed cost-effective (ie, acceptable) on the Y axis (range 0.00-1.00) against various willingness-to-pay (WTP) levels to gain a response on the X axis (range US $0-$500). If there is no WTP to gain an additional response (WTP=US $0), then there is a 14% probability that SbS is acceptable from a cost-effectiveness point of view (because 14% of the simulated ICERs appeared in the SE quadrant). Similarly, the SbS intervention has a 50% probability of being regarded as acceptable if society would be willing to pay US $122 per PHQ-9 response (because ICER=US $122 to gain an additional responder). Assuming a decision-maker would like to have a greater than 50% certainty and instead seeks an 80% probability for acceptability, it follows that the WTP for gaining a treatment responder must be about US $220 (ie, the WTP at which the acceptability curve meets the 80% probability level of acceptability).

#### ICER Response: Societal Perspective

The base case analysis with treatment response as the primary outcome was repeated taking the societal perspective. The incremental effect is as before: a 0.23 greater probability of treatment response with SbS than with EUC but with an incremental societal cost of –$24 (negative cost, hence cost reductions). The fact that societal costs are lower than the health care costs can only be explained by greater productivity of the participants after having received SbS. Absenteeism (and associated costs) may have been reduced because more SbS recipients returned to work than those who received EUC. In addition, a greater proportion of people who received SbS became more productive (ie, less presenteeism).

**Figure 3:**
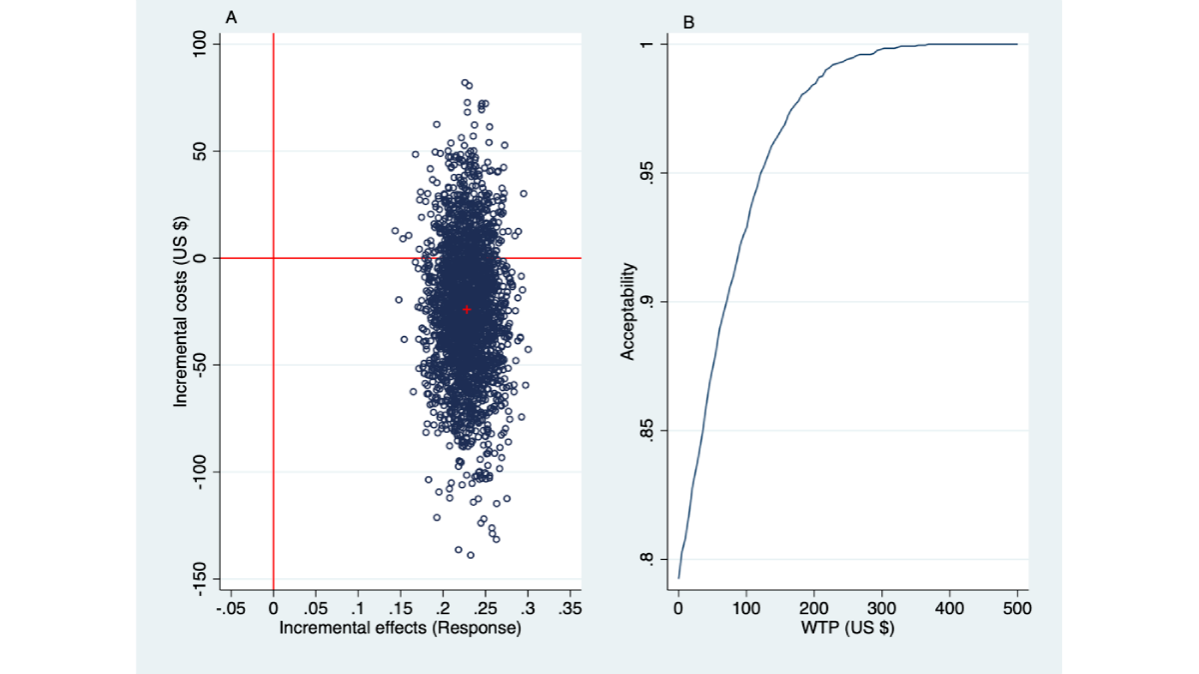
Societal perspective: incremental costs per additional Patient Health Questionnaire (9-item version) responder shown in a (A) cost-effectiveness plane and (B) acceptability curve (2500 bootstraps). WTP: willingness to pay.

The incremental cost per additional treatment responder was also negative (US –$24/0.23 = –$105); therefore, the ICER is said to be “dominant,” because better effects are obtained for less cost with SbS than with EUC. Figure 3 presents the outcomes.

Figure 3A depicts the scatter of simulated ICERs. Of these, 21% appear in the NE quadrant, indicating 21% likelihood that the SbS intervention had better effects albeit for higher costs than EUC. However, a more substantial 79% of the simulated ICERs are in the SE quadrant, which is indicative of a 79% probability that gaining a treatment response with SbS is associated with cost reductions compared with EUC; hence, SbS is deemed “dominant” (ie, represents the more favorable treatment option compared with EUC from a cost-effectiveness point of view). Now, there is no need for a decision maker to review the acceptability curve because, in the context of cost savings, any WTP threshold has become irrelevant. Nonetheless, a look at the acceptability curve (in Figure 3B) shows that, at WTP=US $0, the likelihood of acceptability is 0.78 (78%), increasing to 0.95 (95%) at a WTP of US $110.

In sum, taking the health care perspective, SbS has an 80% likelihood to be regarded as cost-effective when there is a WTP of US $220 for an additional PHQ-9 response. However, taking the societal perspective, SbS is associated with a cost reduction and hence “dominant.” To reiterate, the cost reduction as seen from the societal perspective indicates that people become more productive (lesser absenteeism and presenteeism) after receiving SbS.

#### ICER Remission: Health Care Perspective

**Figure 4:**
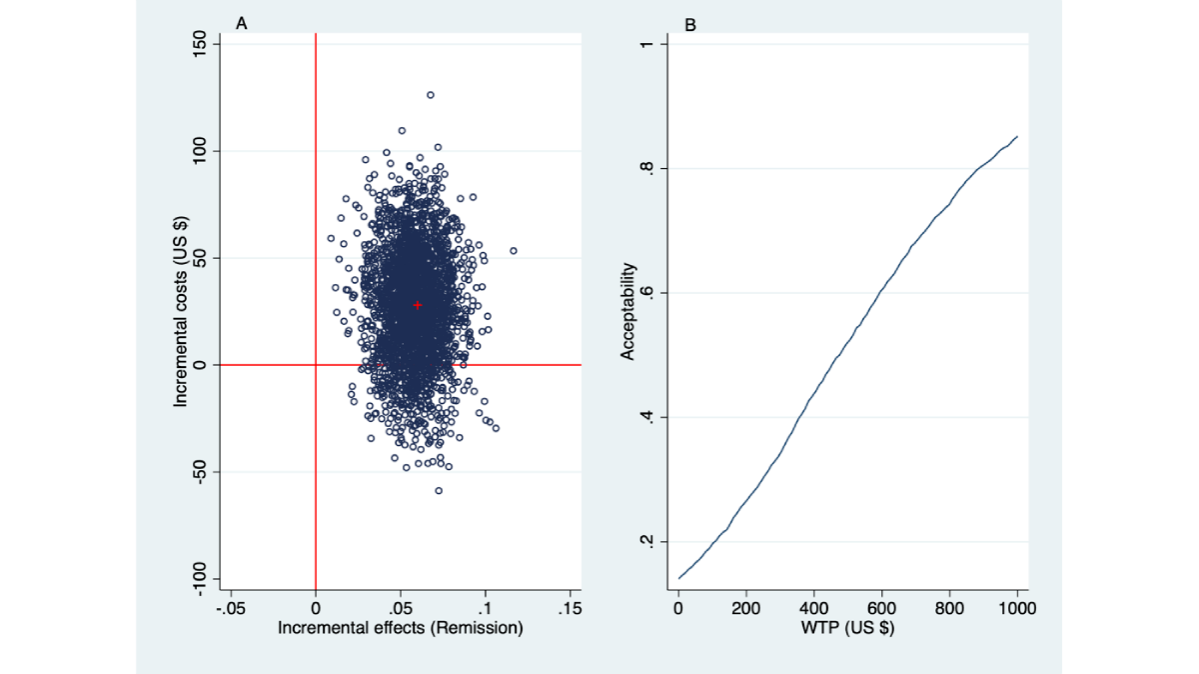
Health care perspective: incremental costs per additional Patient Health Questionnaire (9-item version) responder shown in a (A) cost-effectiveness plane and (B) acceptability curve (2500 bootstraps). WTP: willingness to pay.

The base case analysis was repeated for the secondary outcome, remission. The incremental health care costs remained the same as before, at US $28. The remission rate achieved was higher by 0.058 (95% CI 0.031-0.085) with SbS than with EUC, which was statistically significant (bootstrap SE=0.014, *z*=4.20; *P*<.001). Division of the incremental health care cost by the incremental remission rate gives US $28/0.058=$474. Thus, to achieve a remission with SbS costs more than with EUC, by $474. Figure 4 presents the results.

Since achieving a remission with SbS costs more than with EUC, the acceptability curve needs to be reviewed for decision-making. At WTP=US $0, there is a 14% probability that SbS is deemed to be acceptable from a cost-effectiveness point of view (because 14% of the simulated ICERs appeared in the SE quadrant). At WTP=US $474, the acceptability reached the 50% probability level (because the ICER=US $474 for health care costs per remission). Should a greater than 50% certainty be required for decision-making in health, say, acceptability at the probability level of 80%, then it follows from Figure 4 that the required WTP threshold is about US $840 per remission.

#### ICER Remission: Societal Perspective

Looking at remission and taking the societal perspective, the incremental costs are negative (US –$24), indicating a cost reduction (due to fewer productivity losses after receiving SbS). This implies the SbS intervention dominates EUC as the more cost-effective treatment option as seen from the wider societal perspective.

### Sensitivity Analysis

Table 4 summarizes the outcomes from the base case analysis (incremental costs per response and per remission based on regression imputation) and compares these with those from the sensitivity analysis (MICE using predictive mean matching).

Taking the health care perspective, Table 4 shows that the ICER is US $121 per responder in the base case analysis (US $101 in the sensitivity analysis) and US $474 per remission in the base case analysis (US $141 in the sensitivity analysis).

When taking the societal perspective, SbS was dominant relative to EUC because costs will be saved in both the base case analysis and sensitivity analyses.

Overall, the sensitivity analyses produced results that were fairly similar to the base case analysis but produced a higher remission rate (0.163) relative to the base case analysis (0.058). The estimate of 0.058 can only be interpreted as a small but positive incremental effect of SbS on remission. The estimate of 0.163 is still indicative of a small but positive incremental effect of SbS on remission, and our previous conclusion would not alter in any material way. All in all, the sensitivity analysis produced similar or roughly similar outcomes as the base case analysis. This is important because the study was subject to an expected, but large, dropout; therefore, it is important to assess if the results did not crucially depend on one or another imputation technique but that different imputation techniques indeed produced similar results.

**Table 4:** Base case and sensitivity analyses.

Analysis, perspective, and outcome				Incremental costs (US $)		Incremental effects		ICER^a^ cost (US $) or effect		ICER distribution, %						
										NE		NW		SW		SE
**Base case**																
	**Health care**															
		Response	28		0.228		121		86		0		0		14	
		Remission	28		0.058		474		86		0		0		14	
	**Societal**															
		Response	–24		0.228		Dominant		21		0		0		79	
		Remission	–24		0.058		Dominant		21		0		0		79	
**Sensitivity**																
	**Health care**															
		Response	23		0.228		101		76		0		0		24	
		Remission	23		0.163		141		76		0		0		24	
	**Societal**															
		Response	–29		0.228		Dominant		24		0		0		76	
		Remission	–28		0.163		Dominant		24		0		0		76	

^a^ICER: incremental cost-effectiveness ratio.

## Discussion

### Principal Findings

The health-economic evaluation of SbS versus EUC was conducted from 2 perspectives: the health care perspective and the societal perspective. The evaluation showed that the distinction between both perspectives is important. After all, SbS turned out to be associated with additional costs than EUC when seen from the health care perspective but was associated with cost savings as seen from the more encompassing societal perspective, apparently because the health care costs were more than compensated by the greater productivity of the people who received SbS instead of EUC. To be precise, taking the health care perspective, SbS had an 80% probability to be regarded as cost-effective compared with EUC when there was a WTP of US $220 per additional treatment response or US $840 per additional remission. Taking the more encompassing societal perspective, SbS had a more than 75% probability to be cost-saving while gaining treatment response or remission. Access to evidence-based mental health care in Lebanon is limited due to insufficient resources and infrastructure. Digital mental health interventions offer promising alternatives to address this issue, particularly for vulnerable populations like displaced people and those affected by conflict [11]. In addition, stigma surrounding mental health services discourages individuals from seeking help. To address this, interventions like SbS offer remote support, allowing mental health patients to seek private assistance without fear of negative perceptions [27].

### Limitations

Our study was not without limitations. First, the findings may have been affected by the high dropout rate. Here, it should be noted that high dropout rates were expected, as these are usually associated with digital self-help interventions [11,12,23,28,29]. In the power calculation, we therefore accounted for the high dropout. In addition, different imputation techniques for missing data produced similar results, attesting to the robustness of our findings despite the high dropout rate. Nonetheless, dropout may have influenced the study’s outcomes.

Second, no clinical diagnostic interviews were used to assess depression status. Nevertheless, the PHQ-9 is a reliable instrument and was transformed in clinically relevant metrics such as treatment response and remission using well-established cutoffs.

Third, unit cost prices were mostly based on tariffs and reflected the price levels of the year 2019, which are likely to differ from current price levels in Lebanon where inflation is high.

Fourth, the effects were assessed at 3 months postintervention. Although the effects were maintained over that period, new studies are required to assess longer-term effectiveness and cost-effectiveness of digital self-help interventions and to assess the need to perhaps invite participants to return to SbS, for example after a depressive relapse.

Finally, decision-making based on a health-economic evaluation must consider that there are no universally agreed-on WTP thresholds for gaining a treatment response and remission. Ultimately, it is up to national policymakers what they consider good value for money, which may depend on factors such as the need for health care in the population, possibilities for sustained funding, and likely budget impacts in addition to medical-ethical and equity considerations.

Our study also has some notable strengths. In low- and middle-income countries, resources, expertise, and infrastructure for mental health care research, including health-economic evaluations, are limited [30]. This is one of the larger randomized trials in mental health in a low- to middle-income country and one of the very few studies to assess the cost-effectiveness of a guided, digital self-help intervention for treatment response and remission in such a context. Furthermore, the fact that the study was able to recruit 1249 participants during the COVID-19 outbreak demonstrates the high demand for a low-threshold intervention like SbS.

### Comparison With Prior Work

In terms of clinical outcomes, SbS significantly reduced symptoms of depression, anxiety, and posttraumatic stress and improved functioning [11,12]. These findings were consistent with meta-analytic evidence that digital health interventions for depression offered with some guidance, like SbS, are effective in generating beneficial clinical outcomes [31]. A health-economic evaluation of Syrian refugees in Turkey compared a group-based guided self-help course for stress management with EUC and demonstrated a 97.5% probability of cost-effectiveness at a WTP of US $2802 for gaining a quality-adjusted life year [32].

Regarding the cost-effectiveness of guided digital interventions, Mitchell et al [33] performed a systematic review of 27 economic evaluations of internet-based psychological interventions for anxiety disorders and depression and found that 81% of the internet-based treatments were cost-effective. More recently, Rohrbach et al [34] conducted a systematic review and meta-analysis of 37 trial-based economic evaluations of digital health interventions for people with mental disorders compared with care as usual or “no intervention.” They observed that online therapies for mental disorders were more effective at generating health gains for similar costs as usual care across a range of mental disorders. All in all, our results appear consistent with available evidence in indicating that guided digital self-help has a high probability to be regarded as cost-effective when compared with care as usual. However, it should be noted that the reviews and meta-analyses were based on studies conducted in high-income countries, whereas our study presents evidence from a middle-low income country facing a series of overlapping humanitarian and economic crises.

### Conclusions

The SbS intervention, in addition to having a statistically significant and clinically meaningful effect on depression, anxiety, and stress-related disorders [11,12], had a more than 75% probability of being cost-saving as seen from the societal perspective.

Taking the health care perspective, SbS is associated with additional costs that are acceptable when there is a WTP of US $220 to achieve a treatment responder or US $840 for gaining a remission. In short, this study shows that digital health can be seen as cost-effective or even cost-saving compared with usual care. Apart from its cost-effectiveness, digital care can bring additional benefits because it can be accessed 24/7 from any location without the need to travel to health services and with less fear of stigma. This can be particularly relevant when access to routine care is hampered.

Throughout crises, health care systems are frequently overburdened, the health care workforce health is affected, and access to specialists for support is limited. In such a context, a digital self-help intervention with limited guidance appears to offer a promising and cost-effective approach to respond to humanitarian crises in low-resource settings such as Lebanon.

## Appendix

Multimedia Appendix 1 Mean costs in US $ per participant over time by condition, not including US $26 Step-by-Step costs (n=1249).

## Abbreviations

CHEERS: Consolidated Health Economic Evaluation Reporting Standards
CSRI: Client Service Receipt Inventory
EUC: enhanced usual care
FTC: full-time equivalent
ICER: incremental cost-effectiveness ratio
iMTA: Institute of Medical Technology Assessment
MICE: multiple imputation with chained equations
PHQ-9: Patient Health Questionnaire (9-item version)
SbS: Step-by-Step
SURE: seemingly unrelated regression equations
TiC-P: Trimbos and iMTA Cost questionnaire for Psychiatric illness
WHO: World Health Organization
WTP: willingness to pay

## Data Availability

Deidentified data are available at the open access DANS data repository [35] at [36] and can be acquired through the data management committee of the Faculty of Behavioural and Movement Sciences of the Vrije Universiteit Amsterdam, which can be reached at data.committee.fgb@vu.nl.
