# Supplementary material for: Cost-Effectiveness of Digital Mental Health Versus Usual Care During Humanitarian Crises in Lebanon: Pragmatic Randomized Trial
Source: JMIR Ment Health. 2024 May 29;11:e55544. doi: 10.2196/55544 (PMC11170045; doi:10.2196/55544)
Supplement: Multimedia Appendix 1 [file mental_v11i1e55544_app1.docx]

Multimedia Appendix 1

**Table S1.** Mean costs in $ per participant over time by condition, not including $26 SbS costs (n=1,249)

| **Cost item** | **Group** | **t0** | **t2** | **t2** | **Cum.(t0–t2)** | **(95%CI)** |
| --- | --- | --- | --- | --- | --- | --- |
| . Primary care | EUC | 18.66 | 17.44 | 19.35 | 104.04 | (74.57; 133.52) |
|  | SbS | 17.44 | 20.69 | 15.53 | 100.13 | (77.20; 123.05) |
| . Paramedical care | EUC | .24 | .26 | .23 | 1.39 | (.47; 2.32) |
|  | SbS | .33 | .24 | .18 | 1.23 | (.71; 1.75) |
| . Outpatient care | EUC | 18.08 | 19.00 | 28.46 | 111.91 | (78.95; 144.88) |
|  | SbS | 17.36 | 25.05 | 24.65 | 131.78 | (75.77; 187.79) |
| . Inpatient care | EUC | 4.31 | 8.82 | 0.76 | 32.62 | (0.68; 64.56) |
|  | SbS | 1.81 | 7.43 | .96 | 21.44 | (-3.04; 45.92) |
| . Emergency care | EUC | 2.25 | 2.38 | 1.10 | 9.63 | (2.10; 17.16) |
|  | SbS | 3.39 | 1.66 | 1.01 | 3.21 | (-.35; 6.78) |
| . Pharmacological care | EUC | 1.81 | 1.74 | 1.68 | 8.46 | (6.04; 10.88) |
|  | SbS | 1.44 | 1.18 | 1.15 | 6.19 | (4.11; 8.26) |
| **Total Healthcare** | EUC | 45.35 | 47.77 | 50.57 | 240.63 | (202.70; 278.56) |
|  | SbS | 41.78 | 54.66 | 42.76 | 242.56 | (209.34; 275.77) |
| . work loss days | EUC | 11.81 | 6.17 | 14.69 | 49.25 | (34.78; 63.72) |
|  | SbS | 8.26 | 5.47 | 6.83 | 32.17 | (20.90; 43.44) |
| . work cutback days | EUC | 66.81 | 8.61 | 36.67 | 143.36 | (125.54; 161.18) |
|  | SbS | 56.58 | 8.13 | 22.92 | 111.27 | (94.82; 127.73) |
| **Total Productivity** | EUC | 78.62 | 16.76 | 54.58 | 202.39 | (175.68; 229.11) |
|  | SbS | 64.83 | 15.20 | 31.93 | 150.75 | (129.20; 172.30) |
| **Total Societal** | EUC | 123.97 | 64.53 | 105.15 | 443.02 | (395.84; 490.21) |
|  | SbS | 106.61 | 69.86 | 74.69 | 393.31 | (353.59; 433.03) |
